# How do yeast sense mitochondrial dysfunction?

**DOI:** 10.15698/mic2016.11.537

**Published:** 2016-09-22

**Authors:** Dmitry A. Knorre, Svyatoslav S. Sokolov, Anna N. Zyrina, Fedor F. Severin

**Affiliations:** 1 Belozersky Institute of Physico-Chemical Biology, Moscow State University, Leninskiye Gory 1-40, Moscow 119991, Russia.; 2 Faculty of Bioengineering and Bioinformatics, Moscow State University, Leninskiye Gory 1-73, Moscow 119991, Russia.; 3 Institute of Mitoengineering, Moscow State University, Leninskiye Gory 1, Moscow 119991, Russia.

**Keywords:** mitochondria, yeast, retrograde signaling, ROS

## Abstract

Apart from energy transformation, mitochondria play important signaling roles. In yeast, mitochondrial signaling relies on several molecular cascades. However, it is not clear how a cell detects a particular mitochondrial malfunction. The problem is that there are many possible manifestations of mitochondrial dysfunction. For example, exposure to the specific antibiotics can either decrease (inhibitors of respiratory chain) or increase (inhibitors of ATP-synthase) mitochondrial transmembrane potential. Moreover, even in the absence of the dysfunctions, a cell needs feedback from mitochondria to coordinate mitochondrial biogenesis and/or removal by mitophagy during the division cycle. To cope with the complexity, only a limited set of compounds is monitored by yeast cells to estimate mitochondrial functionality. The known examples of such compounds are ATP, reactive oxygen species, intermediates of amino acids synthesis, short peptides, Fe-S clusters and heme, and also the precursor proteins which fail to be imported by mitochondria. On one hand, the levels of these molecules depend not only on mitochondria. On the other hand, these substances are recognized by the cytosolic sensors which transmit the signals to the nucleus leading to general, as opposed to mitochondria-specific, transcriptional response. Therefore, we argue that both ways of mitochondria-to-nucleus communication in yeast are mostly (if not completely) unspecific, are mediated by the cytosolic signaling machinery and strongly depend on cellular metabolic state.

## INTRODUCTION

In present-day eukaryotes mitochondria play multiple roles such as oxidative phosphorylation, Fe-S clusters biosynthesis, thermogenesis and others (see for review 1,2,3). Some special features of mitochondria make them a unique cellular signaling center. First, mitochondria have two compartments separated from the cytoplasm. Outer membrane is impermeable for molecules with molecular weight above 8 KDa 4, thus the intermembrane space sequesters signaling macromolecules. Indeed, in higher eukaryotes the intermembrane space proteins serve as transducers of programmed cell death activation cascade 5. The list of such proteins includes specific signaling molecules such as Smac 6 and Diablo 6, as well as proteins with well established “day-job” function, e.g. cytochrome *c*, which in higher organisms binds cytosolic Apaf-1 complex to promote apoptosis 7. In yeast, cytochrome *c* was also suggested to have a pro-apoptotic function 8,9, although its cytoplasmic target is still not found. The inner membrane is impermeable for low molecular weight molecules, thus the matrix is able to entrap some metabolic intermediates and ions. Second, mitochondria harbor many enzymes with cofactors capable for reduction of molecular oxygen. This makes mitochondria a potentially powerful source of superoxide and hydrogen peroxide 10,11. Finally, mitochondrial appear to be a natural element of signaling network capable of signal integration. Indeed, mitochondria can converge different inputs by decreasing or increasing the transmembrane potential (e.g. via activation of respiratory chain activity). As the transmembrane potential controls transport of various compounds across mitochondrial membranes (see 12 for review) and also regulates functional states of inner membrane translocators 13, mitochondria can be regarded as an element of signal convergence.

What kind of cellular responses are triggered by mitochondria? As the main mitochondrial function is transformation of energy, one can expect metabolic enzymes to be the central targets of the mitochondrial signaling. Indeed, it was recently shown that overexpression of mitochondrial superoxide dismutase in mammalian cancer cells inhibits AMPK and upregulates glycolytic enzymes via increased flux of hydrogen peroxide 14. Moreover, there are a lot of metabolic enzymes among the targets of retrograde (mitochondria-to-nucleus) signaling cascade mediated by Rtg1/Rtg3 transcription factors (see for review 15). Next, as mitochondria partially rely on their own DNA, mitochondrial DNA damage can cause mitochondrial dysfunction. Indeed, there are several stresses that are more damaging for mitochondrial than for nuclear DNA. An example of such stress is the exposure of yeast cells to anoxia (16; see also 17 for review). In such cases the feedback is required by the nucleus to change the levels of the nuclear-encoded mitochondrial proteins accordingly. It is important to mention here that the nuclei encode most of the proteins localized in mitochondria. Furthermore, a set of changes in mitochondria are required during cell division. Although there are convincing data that in yeast cell cycle arrest does not inhibit replication of mtDNA 18,19, the recent data suggests that mitochondrial biogenesis is thoroughly coordinated with the cell cycle stages 20.

In our review we argue that in yeast the major known routes of mitochondrial signaling are moderated by non-mitochondrial inputs. Despite the importance and complexity of mitochondrial activity, yeast cells, apparently, do not monitor mitochondrial functional state directly. Instead, they monitor important mitochondrially-produced substances, the levels of which also depend on non-mitochondrial factors. The cellular reactions to the imbalances in such substances are also not mitochondria-specific but include modulation of mitochondria-independent processes.

## ATP VERSUS TRANSMEMBRANE POTENTIAL IN RTG-DEPENDENT MITOCHONDRIAL RETROGRADE SIGNALING

Retrograde signaling pathway was originally discovered as a mechanism initiated by mitochondrial dysfunction 21. As a result of its activation, the Rtg3 protein is translocated to the nucleus and activates expression of a set of genes which helps to cope with the dysfunction. In particular, the changes in the expression provide reconfiguration of metabolism aimed to maintain synthesis of vital amino acids (reviewed in 15). One of the possible reasons of mitochondrial dysfunction is exposure of yeasts to specific mitochondrial inhibitors (most of those are produced by bacteria or fungi 22,23). Thus, one of the responses induced by Rtg1/Rtg3 transcription factors is the induction of pleiotropic ABC-transporters expression, that can prevent the delivery of unwanted xenobiotics to mitochondrial targets 24, although the precise mechanism of pleiotropic drug resistance activation is still unknown 15. Rtg2 protein is proposed to be an initiator of this pathway (see reviews 15,25), however, the existence of additional upstream signaling proteins cannot be excluded. Are there any specific Rtg2 ligands responsible for its activation? At least three possible parameters are usually considered as potential hallmarks of mitochondrial dysfunction: alterations in the levels of nucleotide triphosphates, mitochondrial transmembrane potential and reactive oxygen species (ROS, see 26). It was previously shown that introduction of the *ATP1-111* mutation in the cells lacking mitochondrial DNA (*rho0*) increases the mitochondrial transmembrane potential and at the same time prevents expression of the downstream events of the retrograde signaling (i.e. Rtg3-GFP relocalization to the nuclei, 27). This points at the role of the transmembrane potential, although does not address the mechanism of the “sensing”. Conversely, the *in vitro *experiments revealed the role of nucleotide triphosphate binding in activation of Rtg2. It was found that ATP in high concentration induces dissociation of Rtg2 from its downstream target Mks1 28.

On the one hand, these data complement each other. On the other hand, concentration of ATP in the cells does not strictly correlate with mitochondrial transmembrane potential. Under conditions of active glycolytic flux and repressed respiratory chain mitochondria do not contribute significantly to the cellular ATP level 29. Therefore, under such conditions, loss of mitochondrial DNA - the standard way to activate retrograde signaling response - will not necessarily lead to a decrease in cytoplasmic ATP level. Thus, the effect of *Rho0* mutation could be dampened in high glucose concentrations. In agreement with this, it was shown that the level of background retrograde cascade activation is much higher in the cells grown on poor-fermentable carbon sources 30. Moreover, in our hands 31, as well as in the previous high-throughput screen, *rho0 *mutation did not lead to an increase of mRNA of Rtg-targets 32. Finally, the ATP-ase inhibitor oligomycin induces the set of genes that differs from the one activated by *rho0* mutations or uncoupler CCCP 33. This contradiction suggests that Rtg2 signaling depends on ATP level rather than on mitochondrial transmembrane potential.

To summarize, as ATP concentration does not depend on mitochondrial function only, Rtg pathway cannot be regarded as an exclusive mitochondria-to-nucleus signaling line.

## ABERRANT ACCUMULATION OF MITOCHONDRIAL PRECURSORS IN THE CYTOSOL

Taken that Rtg2-mediated signaling is not specific to mitochondrial dysfunction, how do mitochondria provide feedback to the nucleus in case of mitochondrial problems? Higher eukaryotes harbor mechanisms for identification of dysfunctional mitochondria, which is based on impaired protein import 34,35,36. Damaged mitochondria can induce compensatory response 36 or be removed by mitophagy, a mitochondria-specific branch of autophagy 35. In both cases, the mitochondrial dysfunction retards import of specific proteins. In *C. elegance*, transcription factor ATFS-1 has double localization targeting. Inhibition of mitochondrial import induces its relocalization to the nucleus and activation of compensatory response 36. In mammals, a decrease of the transmembrane potential activates mitophagy which relies on PINK and Parkin proteins (see for review 35). *S. cerevisiae *lacks homologs of ATFS-1 or PINK/PARKIN systems. Are yeast cells able to get rid of mitochondria with low transmembrane potential? Although there are several works suggesting the role of mitophagy in yeast mitochondrial quality control 37,38,39, a specific mitochondrial autophagy in yeast is normally induced by starvation 40,41. The latter fact points at the role of mitophagy in maintaining energy and nitrogen balances. Nevertheless, retention of the damaged mitochondria in the mother cell during cell division could ensure their clearance from the growing colony 42,43. We suggested earlier that the presence of such a mechanism could substitute for selective mitochondrial mitophagy 44.

In any case, yeast cells do possess a specialized signaling pathway activated by a drop in the transmembrane potential. Recently it was reported that in yeast, a failure to import mitochondrially-targeted proteins activates mitochondrial precursor over-accumulation stress (mPOS) response, which suppresses the proteotoxic consequences of the precursor accumulation 45. The set of proteins induced acts mainly to reduce the rate of protein biosynthesis. Interestingly, this type of unfolded protein stress, unlike the one caused by the heat shock (reviewed in 46), does not induce accumulation of cytosolic chaperones which act to repair the misfolded proteins. The authors speculate that additional chaperones would not improve the situation: refolding of the cytosolically accumulated precursor proteins could even worsen the situation. Still, the question remains: do mitochondrial precursor proteins bind to a specific signaling ligand in the cytosol or, alternatively, accumulation of non-specific misfolded proteins in the cytosol can trigger mPOS network. The answer to this is not straightforward because conventional stresses causing protein misfolding are not specific to the cytoplasm: heat stress, mutations in the proteasomal genes or major molecular chaperones also cause an increase in proteins folded in the ER (reviewed in 47). At the same time, there were many studies on ectopic expression of hard-to-fold human proteins in yeast: alpha-synuclein, polyglutamine-rich fragments of huntingtin, etc. (see 48,49 for review). Apparently, such expression differs significantly from a general proteostatic stress. Thus, to our knowledge, there are no data on the changes in the proteome caused by exclusively cytosolic bulk protein misfolding.

## AMINO ACIDS-BASED SIGNALING

As a specific mitochondria-to-nucleus signaling based neither on inhibited protein import into mitochondrial matrix nor on mitochondrial transmembrane potential has not been shown so far, a question arises: how yeast cells can measure mitochondrial 'health'? Possibly, the simplest way to monitor mitochondrial state is to measure metabolic intermediates that are produced or modified specifically in mitochondria (see for review 50).

Due to the fact that amino acid (i.e. glutamate and arginine 51,52) biosynthetic pathways are localized in mitochondrial matrix, the cytoplasmic amino acids levels are good candidates for mitochondrial productivity indicators. Indeed, the deficit of glutamine activates Rtg pathway, leading to an increase in transcription of the mitochondrial enzyme Gln1p responsible for its synthesis 53. Interestingly, similar to activation of Rtg by a decrease in ATP concentration, the final step of this pathway’s activation by the drop in the amino acid concentrations also happens in mitochondria-independent fashion. While the molecular mechanism is rather complex 54,55, it was convincingly shown that TOR (target of rapamycin) complex located in the cytosol senses the amino acid deficit and then directly activates Rtg2 protein 56,57.

## RETROGRADE SIGNALING AND REACTIVE OXYGEN SPECIES

Mitochondria are usually considered as a source of reactive oxygen species (ROS). The most common ROS are O_2_•^−^, H_2_O_2_, •OH, NO• and ^1^O_2_. If the level of ROS exceeds the capacities of the defense mechanisms, the cell reaches the state which is often referred to as “oxidative stress”. A precursor of most of the ROS, superoxide anion (O_2_•−), is produced via nonenzymatic reduction of molecular oxygen by electron transport chain components (reviewed in 58,59). Hydrogen peroxide (H_2_O_2_) is produced by dismutation of O_2_•−, and can be reduced fully into water or partially into highly reactive hydroxyl radical (•OH) 60. Some of TCA enzymes also contribute to generation of reactive oxygen species 61. At the same time, mitochondria harbor a robust antioxidant system: for instance, the activity of mitochondrial catalase is several orders of magnitude higher 62 than the maximal rate of hydrogen peroxide production by dysfunctional mitochondria 63. As a result, under normal conditions mitochondria do not export ROS, instead, they can be considered as a sink for them (see 10 for review). However, under stress the capacity of antioxidant systems can be exhausted and the direction of ROS flux can be reverted. For instance, an increase in cytosolic [Ca^2+^] transforms yeast mitochondria into a major source of ROS (see 9 and references within). Moreover, it was shown that Rtg1-Rtg3 signaling pathway plays a hormetic role by increasing mitochondrial ROS production and in this way upregulating antioxidant enzymes 64.

In the states of dysfunction, mitochondria activate signaling to increase the levels of antioxidant enzymes which do not rely on respiratory chain functioning. In particular, it was shown that inhibition of respiratory complex III with myxothyazol induces expression of not only mitochondrial/peroxisomal catalase Cta1 65 but also of cytosolic catalase Ctt1 and of unspecific stress response genes controlled by Msn2/Msn4 transcription factors 65. These data indicate that oxidative stress response induced by mitochondrial dysfunction is general rather than mitochondria-specific. This is in agreement with the data on ethanol-induced oxidative stress: it was shown that high doses of ethanol activate Yap1 66,67, the key cytosolic hydrogen peroxide sensor 68.

## Fe-S CLUSTERS AND HEME

Yeast mitochondria are indispensable for synthesis of such iron-containing compounds as Fe-S clusters and heme. Is the deficit of such compounds perceived by the cells as a manifestation of mitochondrial malfunction? The answer seems to be negative. The signaling pathways initiated under such conditions include the following steps.

First, insufficient levels of either Fe-S clusters or heme induce mitochondria-mediated oxidative stress (reviewed in 69). It is known that Yap1 is the central transcription factor activated by hydrogen peroxide. Interestingly, among other targets Yap1 promotes expression of plasma membrane iron transporters *FET3* and *FET4*, iron regulon gene *FRA2* and *ISU1*, product of which plays a scaffolding role during the assembly of Fe-S clusters 70,71 Hem15, a protein mediating heme biosynthesis, is also among Yap1 targets 70. There is also a specialized transcription factor, Hap1, which is directly activated by heme 72. Importantly, heme synthesis depends not only on functional mitochondria, but also on iron and oxygen availability. At the same time, Hap1 is also sensitive to oxidative stress 73 and is known to induce the expression of mitochondrial and cytosolic genes responsible for respiration and for controlling oxidative damage 74,75,76. Moreover, there is another heme-sensitive transcription factor - protein complex HAP, Heme Activator Protein 77. HAP is the master regulator of the mitochondrial biogenesis in the yeast *S. cerevisiae* 78. It was shown that HAP complex activity is sensitive to ROS signaling and can be restored by an antioxidant as well as by the overexpression of superoxide dismutase Sod1p 79.

Thus, it appears that a general oxidative stress response includes a branch which signals to increase the production of the mitochondrially-synthesized iron-containing molecules. Conversely, the cells upregulate their antioxidant defenses in response to a deficit in the mitochondrially-produced iron-containing substances.

## MITOCHONDRIAL-DERIVED PEPTIDES

Export of Fe-S cluster precursors from mitochondrial matrix in yeasts is mediated by Atm1p, which belongs to the large family of membrane proteins, ABC-transporters 80. Atm1p is partly functionally redundant with the second ABC-transporter localized in mitochondrial inner membrane, Mdl1 81. At the same time, many ABC-transporters are able to transport various substrates with significantly different physico-chemical properties (reviewed in 82). Accordingly it was shown that Mdl1 mediates export of short (6-20 amino acid) peptides, which can be a product of proteolytic degradation of the mitochondrial matrix proteins by Lon protease 83. These peptides (or some of them) are obviously perfect candidates for the role of specific messengers of mitochondria-to-nucleus signaling activated by mitochondrial matrix overload with unfolded proteins. It was shown that the deletion of *MDL1 *gene changes the expression of several nuclear encoded genes under conditions of mitochondria dysfunction induced by the deletion of an important mitochondrial protease *YME1, *while the phenotype of *MDL1 *deletion in the parental cells was much weaker 84. An example of mitochondrial regulatory short peptide was recently discovered in mammalian cells. It was shown that MOTS-c transcript is exported from mitochondrial matrix and translated in cytoplasm, where it activates AMP-dependent kinase 85. Although yeasts do not contain any regions with close homology to MOTS-c, their mitochondrial genome is relatively large and more complex than the human one (human mitochondria harbor shorter DNA, no introns, genes related to oxidative phosphorylation only), meaning that similar mechanisms could still be found in yeasts.

## RETROGRADE SIGNALING AND CELL CYCLE

Mitochondria quantity and quality must be tracked during cell cycle progression, otherwise the daughter or mother cells could inherit insufficient or excessive amounts of the organelles. The former could lead to a complete depletion of mitochondria in some cells and consequent cell death. Indeed, in contrast to the loss of mitochondrial DNA, yeast cells cannot tolerate the loss of mitochondria. To our knowledge, there were no reports describing cases of mitochondria elimination from the wild type yeast cells, although malfunction of mitochondrial transport machinery can induce the formation of buds without mitochondria 86. Thus, it seems likely that mitochondria transmit signal to the nuclei to control cell cycle progression depending on mtDNA and/or mitochondrial proteins abundance.

In 2004 Singh 87 suggested the existence of mitochondria-specific checkpoint, *mitocheckpoint*, which signals to the nucleus upon severe mtDNA damage. Later it was found that growth defects of yeast cells with compromised respiratory activity is due to Rad53-mediated delay of G1- to S-phase transition 88. Recent data also revealed that coordination of nuclear cell cycle progression with mitochondrial biogenesis is regulated at the level of protein import machinery 20. We found that under nitrogen starvation conditions, mitochondria contribute to activation of pseudohyphal growth 31. Such growth is associated with prolonged cell cycle delay in G2-phase 89. We have also shown that signaling mediated by Rtg-proteins contributes to the severity of S-phase arrest induced by telomere dysfunction 90. At the same time, early studies showed that cell cycle arrest does not prevent mtDNA overreplication 18,19. Together, it suggests that although mitochondria influence cell cycle progression and activation of its specific modes (e.g. pseudohypha), mitochondrial signaling branch is integrated together with other signals which influence cell cycle progression.

## CONCLUSIONS

To conclude, beyond their role in energy requirement, mitochondria are recognized as elements of signaling pathways convergence. A plethora of cellular processes rely on their proper functionality which is controlled by a tight cross talk between mitochondria and the nucleus (retrograde signaling) and *vice versa* (anterograde signaling). However, how cells sense mitochondrial functionality or mitochondria signal their status is still unclear and needs a better understanding. Yeast has been widely used as a model to study mitochondrial function for its metabolic features are highly conserved throughout the eukaryotic kingdom.

**Figure 1:**
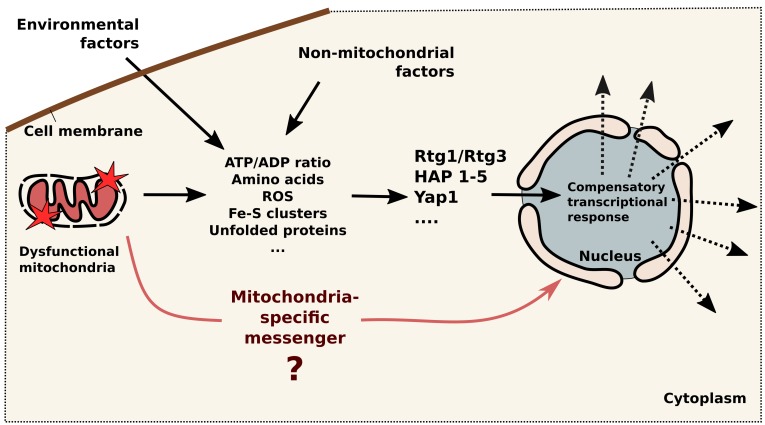
FIGURE 1: Schematic illustration of mitochondria-to-nucleus signaling in yeast. Mitochondrial dysfunction initiate change in concentrations of several factors in the cytoplasm (ATP, amino acids, ROS, Fe-S clusters, unfolded proteins and others), these concentrations also depend on environmental and non-mitochondrial factors. Then factors are detected by the cytosolic sensors (RTG1/RTG3, Hap 1-5, Yap1 and others) which transmit the signals to the nucleus leading to compensatory transcriptional response. Question mark indicates that the direct signaling routes are still not known.

The presented data point that baker’s yeast are devoid of specialized mitochondria-to-nucleus signaling pathways. Instead, mitochondria-initiated cascades are modulated by non-mitochondrial (cytosolic) factors (see Figure 1). Typically, mitochondrial compensatory response is initiated by the changes in concentrations of certain factors in the cytoplasm. Then such problem is detected by the specialized cytosolic sensors which modulate the transcription of the sets of genes (Figure 1). For example, a deficit of glutamate can be caused by malfunctioning mitochondria, by insufficient nitrogen source in the medium or by over-intense protein biosynthesis. The deficit is sensed by TOR complex, which activates Rtg cascade (to improve mitochondrial biosynthetic machinery), invasive growth (to seek nitrogen source) and also slows down the rate of protein synthesis. This does not necessarily mean that the cells are unable to produce transcriptional response which is aimed at mitochondria only. Possibly, a certain combination of changes in the cytosol, e.g. simultaneous drops in the concentrations of ATP and glutamate combined with mild oxidative stress, can induce transcriptional changes mainly affecting mitochondria. Also, it is still possible that the direct signaling routes, similar to mammalian MOTS-c - dependent pathway, do exist in yeast. In our opinion, it is likely that mPOS network is initiated by the specific precursors (as opposed to bulk misfolded protein). If so, such precursor can be considered as a classical signaling intermediate. Short peptides exported by mitochondrial ABC-transporter Mdl1 are also candidates for the role direct signaling molecules.
